# The Role of Vesicular Stomatitis Virus Matrix Protein in Autophagy in the Breast Cancer

**DOI:** 10.31557/APJCP.2021.22.1.249

**Published:** 2021-01

**Authors:** Fatemeh Sana Askari, Alireza Mohebbi, Abdolvahab Moradi, Naeme Javid

**Affiliations:** 1 *Student Research Committee, School of Medicine, Golestan University of Medical Sciences, Gorgan, Iran. *; 2 *Stem Cell Research Center, School of Medicine, Golestan University of Medical Sciences, Gorgan, Iran. *; 3 *Department of Microbiology, School of Medicine, Golestan University of Medical Sciences, Gorgan, Iran. *

**Keywords:** Vesicular stomatitis virus, matrix protein, breast cancer, beclin-1, autophagy

## Abstract

**Background:**

Breast cancer is one of the most difficult malignancies to treat. Therapeutics is used to target and kill the cancer cells. Non-human oncolytic viruses have the ability to cause cell death directly to cancers. The objective here was to investigate the role of Vesicular Stomatitis Virus (VSV) Matrix (M) protein in autophagy in the breast cancer cell line.

**Methods:**

Two different VSV wild type and mutant (M51R) M protein constructs were produced. Breast cancer cell line BT-20 was transfected by either wild type or mutant vectors. Transfection efficiency was measured using a fluorescent microscopy. Expression of VSV M protein was investigated at protein level. Cell cytotoxicity was measured using an MTT assay. The autophagy pathway was studied by Beclin-1 immunoassay. Data were statistically analyzed between different transfected groups.

**Results:**

It has been shown that the VSV M protein induced higher levels of Beclin-1 than the M51R mutant in the BT-20 cell line. Increased levels of Beclin-1 were also associated with VSV M cell-induced cytotoxicity.

**Conclusion:**

It has been shown here that VSV wild type or mutant M proteins can cause autophagy-induced cell death by increasing Beclin-1 expression. This includes the possible role of VSV to be used as an oncolytic virus in breast cancer treatment.

## Introduction

Different treatments have been used to defeat cancer cells. However, with the discovery of oncolytic features of certain viruses or oncolytic viruses (OVs), researchers have drawn attention to the use of these viruses to destroy cancer cells (Bridle et al., 2013). OVs are multi-mechanistic antitumor agents that directly infect and destroy cancer cells (Kelly and Russell, 2007). OVs cause oncolysis directly via apoptosis, necrosis, pyroptosis, and autophagy (Abou El Hassan et al., 2004; Whilding et al., 2013). Tumor cells with Defective Interferon Response and Defects in Ras, p53 or Myc signaling pathways have also been shown to be susceptible to VSV replication (Balachandran et al., 2001; Stojdl et al., 2000). There are a variety of recognized viruses with oncolytic abilities. One of these viruses, ONCORINE, is now approved for cancer treatment (Garber, 2006). Talimogene laherparepvec (T-VEC), a genetically modified herpes virus, was another viral drug used to treat advanced melanoma (Cho and Kwon, 2012). More viruses, including Newcastle Disease Virus ( NDV), Parvoviruses, Myxoma virus, Reovirus, Seneca Valley Virus have an inherent oncolytic capability (Liu and Kirn, 2007). Some genetically modified viruses such as Vesicular Stomatitis Virus (VSV) are also potent to be used for cancer oncotherapy. 

VSV is a fast-replication, negative-sense RNA virus. A natural wide range of VSVs and its high malignant cell tropism, rare pre-existing immunity, simple biological structure, and weak or no human pathogenicity are the main factors that make VSV an appropriate option for oncolytic cancer virotherapy (Balachandran and Barber, 2000). VSV comprises five proteins, namely nucleocapsid (N), matrix (M), phosphoprotein (P), glycoprotein (G), and broad polymerase (L) proteins (Georgel et al., 2007). M protein plays a crucial function in the translation of viral proteins within infected host cells (Mire and Whitt, 2011). However, M protein is known to direct the oncolytic process to the tumor site. Studies have shown that cancer cells are more prone to VSV containing the substituted M protein (M51R) (Hastie et al., 2013). The mutant strain of VSV was thus introduced to be used in therapeutic approaches.

Further studies indicate the safety of oncolytic use of VSV in the treatment of cancer cells. As potent inducers of interferon type 1 response, VSV R51M mutants are not capable of replicating within normal cells (Obuchi et al., 2003). The first analysis of M51R was based on a review of the temperature sensitive (ts) strain of VSV, Ts082, grown on the BHK-21 cell line. Coulon et al.’s research has shown that VSV containing M51R substitution is capable of shutting down the cellular RNA synthesis machine and for effective viral transcription (Coulon et al., 1990). In another study by Hastie, et al, VSV-ΔM51 was shown to be capable of infecting pancreatic adenocarcinoma (PDA) cell lines in the mouse. This recombinant virus has been able to replicate and destroy cancer cells in Vivo (Hastie et al., 2013). These data indicate that VSV M mutant can lead to cancer cells through any of the mechanisms behind inducible cell dead. Autophagy is one of the essential mechanisms by which VSV can cause cell death.

Autophagy is a homeostatic cell recycling process that helps to destroy damaged cellular proteins and organelles in all living cells (Zakeri et al., 1995). The role of autophagy in cancer is controversial (Malilas et al., 2014; Nabizadeh et al., 2016; Olagnier et al., 2017). Becline-1 is a coiled-coil protein and an essential primary autophageal molecule (Liang et al., 1998). The *becline-1 *gene was found to be inhibited in 75% of ovarian, 50% of breast and 40% of prostate cancer (Aita et al., 1999). Beclin-1 expression has been reported to be different in various cell lines (Gozuacik and Kimchi, 2004; Liang et al., 1999). NF-kB signaling is involved on the autophagy pathway. In a study by Shulak et al, NF-kB inhibition was found to inhibit VSV replication and subsequently inhibit cancer cell death (Shulak et al., 2014). In primary chronic lymphocytic leukemia (CLL) cells after VSV infection, the mechanistic role of autophagy in mediating cell death has been shown (Samuel et al., 2013). In addition, it was found that VSV induces apoptosis accompanied by autophagy through strong activation of caspase-3 (Schache et al., 2009). Overall, more evidence must be given for the role of VSV-induced autophagy in cancer-cell death.

The goal here was to investigate whether a construct expressing M51R mutant is capable of inducing cancer-cell death in the breast cancer cell line, BT-20, via autophagy. 

## Materials and Methods

*Cell culture and VSV preparation*

BT-20 human breast cancer cell line was purchased from the Iranian Biological Resource Center (IBRC, Tehran, Iran). The cell line was propagated in tissue culture flasks with complete medium containing Dulbecco’s modified Eagle medium (DMEM-F12; Gibco) supplemented with 10% fetal bovine serum (FBS; Gibco), 100μg/ml streptomycin (Gibco), which atmosphere containing 5% CO_2 _at 37 °C. The reagents were provided from the previous study (Baghban Rahimi et al., 2018; Mohebbi et al., 2019). The medium was replaced two times per week with fresh complete medium. After reaching to >90% confluence, cells were harvested and seeded for transfection. A VSV was kindly provided by Dr. Hadi Razavi Nikoo. The virus with multiplicity of infection (MOI) 5 was used as a positive control for western blot and ELISA. 

*Plasmids and transfection*

VSV M wild type and R51M mutant construct (pcDNA3.1-wt and pcDNA3.1-M51R) provided from the previous study (Douzandegan et al., 2017). After optimization, BT-20 was transfected with either pcDNA3.1-wt or pcDNA3.1-M51R lipofectamine2000TM reagent (Invitrogen, USA), 3 × 10^4^ cells/well were seeded onto 96-well plates containing complete medium. After confluence reached to >90%, 100 ng of each construct along with 2 µl of lipofectamine2000TM reagent were used for transfection. Briefly, the constructs and lipofectamine2000TM reagent were diluted with antibiotic-free DMEM-F12 separately and placed at room temperature for 10 minutes. Then constructs mixed with lipofection reagent at final volume of 50 μl. The mixture was stored at 37°C for 45 minutes. After 45 minutes, mixtures were added onto the cells. Cells were kept at 37°C with 5% CO_2_ for 4 hours. Ultimately, the transfection solution was aspirated and replaced with 100 μl of complete medium. 24 hours later cells were screened for transfection efficiency by fluorescence microscopy (Olympus BX51, London, UK).

*Infectivity assay*

3 × 10^4^ cells/well were seeded into a 96-well plate. Cells were exposed to eitherpcDNA3.1-wt, pcDNA3.1-M51R, or empty vector. For each construct, two concentrations (100 and 200 ng) were used. The cell viability was assessed by adding 3-(4, 5-dimethyl-2-thiazolyl)-2,5-diphenyl-2H-tetrazolium bromide (MTT) to transfected wells after 24hr, 48hr, and 72hr post transfection. Each test was repeated for three time in triplicate. After 3 to 4 hours later, the supernatant was discarded and Formazan crystals were dissolved in Dimethyl sulfoxide (DMSO). Thirteen minutes later, the optical density (OD) was read at 570 nm.

*Western blot analysis*

106 BT-20 cells were seeded into a 6-well plate. 24hr after propagation, cells were transfected with either pcDNA3.1-wt, pcDNA3.1-M51R, or pcDNA3.1 empty vector. Each test performed in duplicate. 24 hr later cells were lysed with lysis buffer (Tris 50 mM, NaCl 150 mM, %1.0 Triton x-100). Proteins were resolved by sodium dodecyl sulfate-polyacrylamide gel electrophoresis (SDS-PAGE) on 12% polyacrylamide gels, then gels were electro-blotted onto a nitrocellulose membrane and blocked with 3% bovine serum albumin (BSA). The membrane was washed (PBS+Tween-20) 5 times. The membrane was then incubated with primary mouse anti-VSV M monoclonal antibody (Kerafast, USA) at 4ºC overnight. Next day membrane washed as the same. Next, HRP-conjugated Goat-anti-mouse IgG secondary antibody was inoculated to the membrane. Bands were observed by using Electro Chemo-Luminescence (ECL; Santa Cruz Biotechnology Inc, USA).

*Human Beclin-1immuno assay*

3 × 10^4^ BT-20 human breast cancer cells were grown in 24-well plate as explained above. Cells were transfected with either pcDNA3.1-wt or pcDNA3.1-M51R. Cells were lysed by sonication at 24 hr, 48 hr, and 72 hr post transfection. Experiments were performed in duplicate. Beclin-1 activity was determined by using Sandwich ELISA (LifeSpan BioScience Inc., USA) according to the manufactured protocol (Life Span Biosciences). Briefly, standard sample was diluted and read at 450 nm to make a standard curve. Concentration of Beclin-1 in test or control samples was calculated according to the standard. Furthermore, a mock cell and VSV infected BT-20 were used as a negative and positive controls, respectively.

*Statistical analysis software*

Graphs and statistics were performed using Microsoft office Excel and GraphPad Prims v7. For statistical analysis, Tuckey, t-test and ANOVA tests were used. The p-values of less than 0.05 were considered as statistically significant.

## Results

*Optimization of BT-20 breast cancer cell line transfection using lipofectamine 2000*

**Figure 1:**
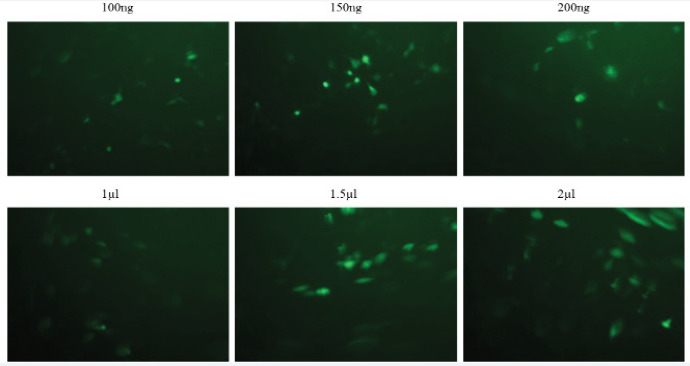
BT-20 Transfection Set-up. Upper row shows three different concentrations of GFP/pcDNA3.1+ vector and lower row demonstrates three volume of Lipofectamine 2000. Only 100ng of GFP/pcDNA3.1+ plasmin was used with Lipofectamine 2000 dilutions

The efficient and successful transfection was achieved at 100ng/µl plasmid 2μl lipofectamine (Figure 1).

**Figure 2:**
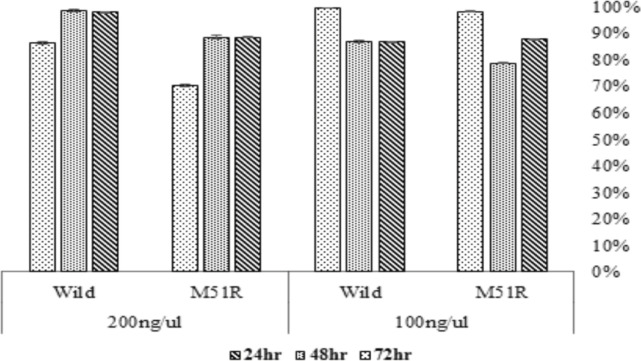
Cytotoxicity of VSV M Wild Type and M51R Mutant in Transfected BT-20 Cell Line

MTT assay was performed to evaluate the cytotoxicity induced by VSV M wild type and VSV M M51R mutant. As it is illustrated in Figure 2, both wild type and M51R mutant of VSV M protein induced cell cytotoxicity. The observed cytotoxicity was not significantly different. However, there was a significant (p-value < 0.001) difference between two pcDNA3.1-wt and pcDNA3.1-M51R at 48hr post transfection. 

**Figure 3:**
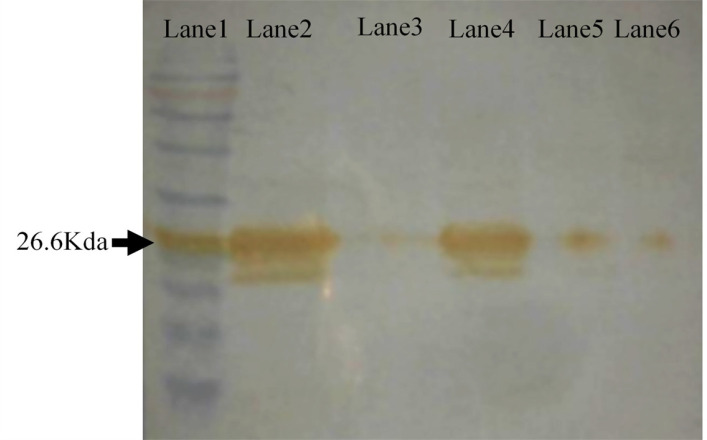
Western Blot Analysis of VSV M Protein. The specific bands are illuminated by using DAB. Lane 1 is protein ladder 240kda (Sinaclon, Tehran, Iran). Lane 2 is the virus infected cell line. Lane 3 shows is mock. Lane 4 is a positive viral control. Lanes 5 and 6 are transfected cell lines with plasmid containing wild type and M51 mutant, respectively

The expression of the VSV M protein was further evaluated by using western blot. In this assay, BT-20 was infected with VSV virus at 5 moi. Figure 3 shows a band with ~26.6Kda size corresponding to VSV M protein.

**Figure 4:**
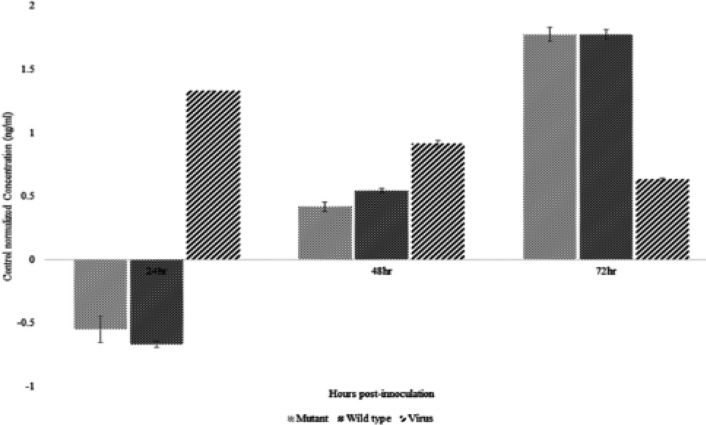
Control-Normalized Levels of Beclin-1 in BT-20 Transfected with Either VSV virus, pcDNA3.1-wt, or pcDNA3.1-M51R. No detectable levels of Beclin-1 was observed at 24hr post inoculation with plasmids codding M proteins. This is partly because of cellular stress induced by plasmids and cell death

A quantitative sandwich ELISA with specificity ranging from 0.156 ng/ml to 10 ng/ml was used for quantification of Beclin-1. A standard curved has been constructed (R2=0.99) according to the provided instruction. Figure 4 shows control-normalized levels of Beclin-1 in BT-20 inoculated with either VSV virus, pcDNA3.1-wt, or pcDNA3.1-M51R at three different times 24, 48, and 72 hr. Data were analyzed by Tukey test. No detected levels of Beclin-1 has been observed in BT-20 transfected with pcDNA3.1-wt or pcDNA3.1-M51R at 24 hr post inoculation. Interestingly, BT-20 infected with VSV virus has been shown gradually decreased levels of Beclin-1. There was no significant difference between Beclin-1 concentrations between pcDNA3.1-wt and pcDNA3.1-M51R at 72 hr post transfection. In correlation with that was observed in cytotoxicity assay, increased levels of Beclin-1 was observed in cell transfected with pcDNA3.1-wt than that in pcDNA3.1-M51R at 48hr post transfection (p-value < 0.05).

## Discussion

Breast cancer is one of the most common cancers among women in the world and is still on the grow (Hortobagyi et al., 2005). There are few common treatments against breast cancer, including surgery, chemotherapy (Greco and Marotti 2006), radiotherapy (Walker et al., 2019), immune therapy (Heimes and Schmidt, 2019), and targeted therapy (Li et al., 2019). Despite a number of therapies, it has been shown that they have not been effective and that there are deaths among patients every year. New therapeutic approaches must also be tested. Oncolytic virotherapy using non-human viruses is a new therapeutic strategy used for cancer treatment. Various studies have focused on the therapeutic use of Vesicular Stomatitis Virus (VSV) in various cancers. Thus, it has been shown that VSV is effective in the treatment of cancers, such as endometrial cancer (Liu et al., 2014), malignant melanoma (Blackham et al., 2013), pancreatic adenocarcinoma (Blackham et al., 2014), glioblastoma multiform (Cary et al., 2011), neuroendocrine tumor (Randle et al., 2013), Head and neck squamous carcinoma (Porosnicu et al., 2009), hepatocellular carcinoma (Shinozaki et al., 2005), prostate cancer (Ahmed et al., 2004), hematologic malignancies (Lichty et al., 2004), and colorectal cancer (Stewart et al., 2011). The effectiveness of this virus in the treatment of breast cancer was also tested on different cell lines of the breast cancer (Le Boeuf et al., 2017; Ebert et al., 2005). 

It has been shown that deletion of methionine 51 in the VSV matrix protein improves its tumor specificity and prevents its involvement in normal cells with healthy immune protection (Lun et al., 2006). Therefore, we investigated in this study the ability of constructs expressing VSV wild type and VSV matrix protein mutant M51R to induce cancer cell death by triggering the autophagy pathway. 

The toxicity was measured at different time points by MTT in the BT-20 cell line transfected with 100 ng and 200 ng pcDNA3.1-wt or pcDNA3.1-M51R. The findings showed that at 12 hr, 48 hr, and 72 hr post transfection, two proteins had high toxicities (>70%) at all concentrations. These results were consistent with previous studies, which demonstrated the toxicity of both mutant and wild M proteins in the cancer cell lines in concern (Stojdl et al., 2003). The mutant M protein has been shown to have more propensity to interferon-deficient cancer cells than the wild type (Lun et al., 2006; Stojdl et al., 2003). Interestingly, the toxicity caused by wild type M protein at two concentrations at any given time was significantly higher than that caused by M51R mutant. This means that of VSV M51R mutant can be used for oncolytic virotherapy in BT-20 cell line. 

Since both concentrations had the same effects, further assays were performed using 100 ng/μl. Whether or not cell death occurred because of the autophagy pathway, the level of Beclin-1 analyzed in the transfected cells. At 24 hours after transfection the expression of Beclin-1 was undetectable in the transfected cells. In the mean time, Beclin-1 was overexpressed in the VSV inoculated cell line. This was partly due to either plasmids or lipofection reagent-induced cell death. The Beclin-1 expression, however, was restored at 48hr and 72hr post transfection. At 48 hr an increased expression was observed in Beclin-1 and reached its peak at 72 hr post-transfection. The amount of Beclin-1 was, however, decreased at the next two points in time. This is due to cell death caused in pathways other than autophagy by the virus (Cary et al., 2011).

These results are also consistent with other studies. Nguyen et al, demonstrated that induction of the Nuclear factor erythroid 2-related factor 2 (Nrf2) signal is needed to replicate VSVD51 and oncolysis in both in vitro and in vivo (Nguyen et al., 2009). In another study, Shulak et al, showed NF- κ B activity through RELA/p65 signaling modulation, causing autophagy induction, enhancing VSV replication, and eventually causing cancer cells to die (Shulak et al., 2014).

Autophagy is a very complex process. The role of autophagy in cancer therapy is controversial, despite the numerous studies performed in this area. Dependent on the stage of cancer, autophagy seems to be geared towards a prophylactic approach to tumor suppression (Santana-Codina et al., 2017). There is evidence that autophagy performs a tumor suppressor through its regulatory Beclin-1 protein, which has an anti-tumor role (Gong et al., 2013). Previously, the significance of autophagy and particularly of Beclin-1 activity has been shown (Liang et al., 1999). It has been shown that 40-75% of sporadic human breast cancers in the *Beclin-1* gene region have mono-allelic deletion (Aita et al., 1999; Saito et al., 1993). The present study provides further evidence of the significance of autophagy and Beclin-1 to cause cell death in the breast cancer cell line BT-20.

Here, BT-20 was used as a breast cancer cell line. It should be noted that the cell line is very slow growth (63 hr duplication time) and chemotherapy-resistance. However, for the first time we report the autophagy induced with VSV and its M51R mutant and wild type M proteins in that cell. The Beclin-1 concentrations were evaluated in this analysis. However, evaluation of the other approaches leading to an increase in autophagy is warranted (Olagnier et al., 2017; Shulak et al., 2014; Tallóczy et al., 2002).

In conclusion, here, both wild type VSV and mutant M proteins have been shown to be able to kill breast cancer cells via the autophagy pathway. This suggests the possible role of the VSV M protein in the treatment of breast cancer for use as an oncolytic virus. With the evidence given here, VSV M proteins can be used to induce cell death in the chemotherapy-resistant cell lines.
